# Bubbles in 2D Materials: Formation Mechanisms, Impacts, and Removal Strategies for Next-Generation Electronic Devices

**DOI:** 10.3390/nano15241888

**Published:** 2025-12-16

**Authors:** Kaitai Du, Baoshi Qiao, Xiaolei Ding, Changjin Huang, Huan Hu

**Affiliations:** 1 ZJU-UIUC Institute, International Campus, Zhejiang University, Haining 314400, China; 2 State Key Laboratory of Silicon and Advanced Semiconductor Materials, Zhejiang University, Hangzhou 310027, China; 3 School of Mechanical and Aerospace Engineering, Nanyang Technological University, Singapore 639798, Singapore; cjhuang@ntu.edu.sg; 4 State Key Laboratory of Fluid Power & Mechatronic Systems, Zhejiang University, Hangzhou 310027, China

**Keywords:** two-dimensional materials, vdW heterostructures, bubbles, regulating interface

## Abstract

Two-dimensional materials and their van der Waals heterostructures have shown great potential in quantum physics, flexible electronics, and optoelectronic devices. However, interfacial bubbles originated from trapped air, solvent residues, adsorbed molecules and reaction byproducts remain a key limitation to performance. This review provides a comprehensive overview of the formation mechanisms, characteristics, impacts, and optimization strategies related to bubbles in 2D heterostructures. We first summarize common fabrication approaches for constructing 2D heterostructures and discuss the mechanisms of bubble formation together with their physicochemical features. Then, we introduce characterization techniques ranging from macroscopic morphological observation to atomic-scale interfacial analysis, including optical microscopy, atomic force microscopy, transmission electron microscopy, and spectroscopic methods systematically. The effects of bubbles on the mechanical, electrical, thermal, and optical properties of 2D materials are subsequently examined. Finally, we compare key interface optimization strategies—such as thermal annealing, chemical treatments, AFM-based cleaning, electric field-driven approaches, clean assembly and AI-assisted methods. We demonstrate that, although substantial advances have been made in understanding interfacial bubbles, key fundamental challenges persist. Future breakthroughs will require the combined advancement of mechanistic insight, in situ characterization, and process engineering. Moreover, with the rapid adoption of AI and autonomous experimental platforms in materials fabrication and data analysis, AI-enabled process optimization and real-time characterization are emerging as key enablers for achieving high-cleanliness and scalable van der Waals heterostructures.

## 1. Introduction

**Figure 1:**
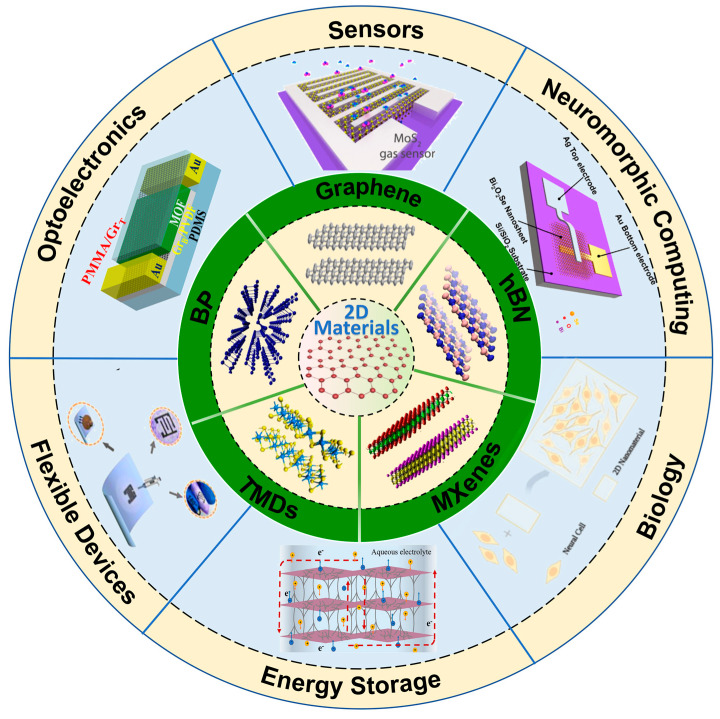
Two-dimensional Materials and their applications (reproduced with permission from [29,30,31,32,33,34,35,36,37,38,39,40]).

The discovery of graphene has stimulated extensive exploration into a broad family of two-dimensional (2D) materials. To date, researchers have identified numerous 2D atomic-layer systems, including transition metal dichalcogenides (TMDs), hexagonal boron nitride (hBN), black phosphorus (BP), and MXenes [1,2,3,4,5,6]. By controllably stacking these atomically thin layers, van der Waals (vdW) heterostructures can be constructed, where the resulting interfaces serve as key platforms for investigating exotic quantum phenomena and developing high-performance devices [2,7,8]. Currently, 2D materials and their heterostructures have demonstrated promising applications in sensors, optoelectronics, flexible electronics, energy storage, biomedicine, and neuromorphic computing (Figure 1) [9,10,11,12,13,14,15,16].

Despite remarkable advances in the fabrication of 2D materials and vdW heterostructures, interfacial cleanliness remains one of the critical bottlenecks limiting device performance. During fabrication, contaminants, adsorbed molecules, and residual gases are inevitably trapped between layers [17]. These interfacial residues tend to aggregate and form nanoscale-to-microscale bubbles, resulting in localized lattice distortion and degraded carrier transport, among other detrimental effects [18]. Therefore, effective removal of bubbles in vdW heterostructures is crucial for achieving high-performance devices. In recent years, significant progress has been made toward understanding the formation and evolution of interfacial bubbles [19], leading to a variety of removal strategies including thermal annealing, chemical-assisted cleaning, and atomic force microscopy (AFM) scanning [20,21,22,23,24,25]. Meanwhile, the rapid emergence of artificial intelligence (AI) in materials preparation, imaging analysis and autonomous experimental platforms provides new opportunities for diagnosing interfacial contamination and optimizing bubble removal conditions [26,27,28]. However, a systematic understanding of the origin, characterization, impact, and elimination mechanisms of interfacial bubbles remains lacking.

In this review, we provide a comprehensive summary of bubbles in 2D vdW heterostructures. We first discuss their formation mechanisms, followed by a summary of commonly employed characterization techniques. We then elaborate on the influence of bubbles on the mechanical, electrical, thermal, and optical properties of 2D materials. Subsequently, we compare different bubble removal methods in terms of their working principles and applicability. Finally, we discuss the current challenges in the field and outline future directions toward achieving atomically clean, defect-free vdW interfaces, with particular attention to how AI-enabled process optimization and imaging analysis may reshape future interface engineering and extend bubble-cleaning strategies toward wafer-scale integration.

## 2. Formation and Characteristics of Interfacial Bubbles

The previous section highlighted the importance of 2D materials and their vdW heterostructures, as well as the critical challenges posed by interfacial bubbles. To understand why bubbles are prevalent and severely detrimental to device performance, it is essential to trace their origin within typical fabrication workflows. Before discussing bubble characterization, impact, and removal strategies, this section reviews the major synthesis routes of 2D materials and stacking techniques used to construct heterostructures, laying the foundation for understanding bubble formation mechanisms.

### 2.1. Fabrication of 2D Materials

**Figure 2:**
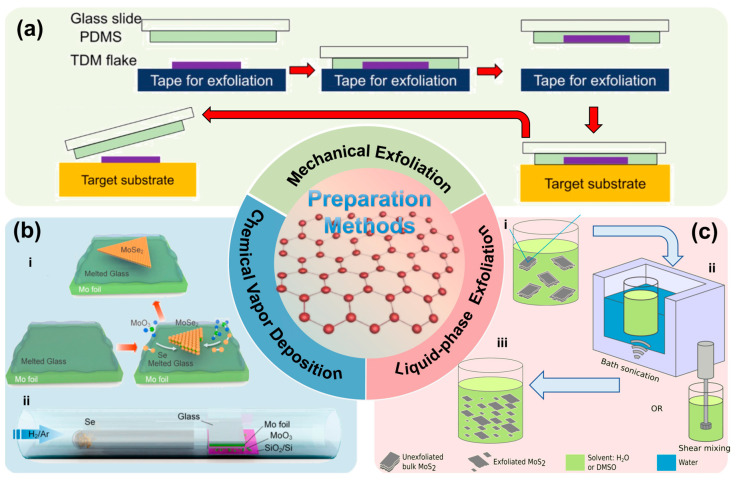
Two-dimensional materials preparation methods (**a**) Schematics of the mechanical exfoliation method; (**b**) Schematic of CVD growth of 2D MoSe_2_, i. Low-nucleation growth enabled by molten glass, ii. CVD growth setup on molten glass, (Reproduced with permission from [56]); (**c**) Schematics of LPE of 2D MoS_2,_ i. Dispersion of MoS_2_ in solvent, ii. Liquid-phase exfoliation via shear mixing or ultrasonication, iii. Final MoS_2_ nanosheet dispersion (Reproduced with permission from [57]).

2D materials were initially obtained via mechanical exfoliation. The typical procedure involves selecting high-quality layered crystals, repeatedly pressing and peeling using polyimide (PI) tape or polydimethylsiloxane (PDMS) substrates to obtain thin flakes, transferring the flakes onto target substrates, and identifying monolayer regions using optical microscopy, AFM, or Raman spectroscopy [41,42,43]. Figure 2a illustrates a representative process. Owing to its simplicity, low cost, and ability to produce high-quality crystals, mechanical exfoliation remains widely used in fundamental research.

Chemical vapor deposition (CVD) represents another important strategy for scalable synthesis of high-quality 2D materials. In this method, transition metal or metal catalyst substrates are placed inside a reaction chamber, precursor gases are supplied under elevated temperatures and controlled atmospheres, and monolayer or few-layer films form through nucleation and surface reactions [44]. In 2009, Ruoff et al. reported the first successful CVD synthesis of monolayer graphene on copper foils [45], followed by large-area monolayer MoS_2_ growth by Lee et al. [46] and monolayer hBN by Kim et al. [47]. The CVD method is illustrated in Figure 2b and has become the mainstream pathway toward industrial production.

Liquid-phase exfoliation (LPE) is another scalable route. Bulk layered crystals are dispersed in appropriate solvents or surfactant-containing aqueous solutions and exfoliated via ultrasonication, shear, or electrochemical intercalation to overcome vdW interactions, followed by centrifugation and filtration to obtain flakes of controlled thickness [48]. Pioneering work demonstrated high-yield monolayer graphene dispersions via ultrasonication in organic solvents [49], later generalized to a wide range of layered compounds [50]. Figure 2c shows the LPE process.

Recent advances in AI have significantly improved these traditional fabrication methods. Machine learning (ML)-assisted approaches can optimize CVD synthesis parameters to achieve better control over crystal morphology and defect density [28,51,52,53], enable automated identification and classification of exfoliated flakes in mechanical exfoliation [54], and predict size distributions in liquid-phase exfoliation through sparse modeling techniques [55]. This integration of AI transforms empirical trial-and-error processes into data-driven, rationally designed fabrication strategies, substantially enhancing the quality and reproducibility of 2D materials preparation.

### 2.2. Construction of vdW Heterostructures and Bubble Formation

After synthesis, 2D layers are assembled into heterostructures through stacking. Common methods include dry transfer, wet transfer, and in situ CVD growth. Regardless of method, weak interlayer interactions and surface energy mismatches make bubble formation highly likely during transfer and lamination [19,58,59].

Dry transfer relies on polymer stamps to pick up and release 2D flakes [43]. The typical process is as follows: high-quality single crystals are first obtained via mechanical exfoliation; subsequently, layer pick-up and precise positioning transfer are achieved through controlled temperature, stress, and surface energy; finally, the layers are released onto the target substrate to form heterostructures. The dry-transfer method offers advantages such as operational flexibility, smooth interfaces, and the ability to control twist angles [60]. During transfer, trapped air, moisture, and polymer residues often form sealed cavities, stabilizing high-pressure bubbles that distort the stacked interface and degrade electronic performance [17,18,61].

The wet transfer method typically involves solvent- or aqueous-assisted separation and redeposition of 2D films and is commonly used for transferring large-area CVD-grown graphene, hBN, or TMD films [45]. The typical process includes: spin-coating a support layer such as polymethyl methacrylate (PMMA) or thermal-release tape on the growth substrate; etching the metal substrate using an etchant; transferring the floating 2D film through a water bath; and finally, transferring it onto the target substrate and removing the support layer [62]. This method is suitable for large-area fabrication; however, solvent molecules and ionic residues are inevitably introduced during etching and rinsing, and the residual water layer at the interface can evaporate during subsequent annealing, easily forming bubbles and, in severe cases, causing interlayer delamination [63].

In addition to transfer-based assembly strategies, in situ continuous growth via CVD can directly form vertical or lateral heterojunctions on a single substrate, avoiding interface contamination introduced by mechanical transfer. By controlling the temperature, precursor supply sequence, and substrate surface activity, controllable growth of heterostructures such as graphene/hBN and WS_2_/MoS_2_ can be achieved [47,64]. This method generally yields cleaner interfaces; however, during in situ growth, nanoscale bubbles or cavities may still form due to uneven nucleation density, substrate surface roughness, or the release of reactive gases, particularly when there are large deviations at bilayer interfaces or abrupt changes in growth rate [65]. Moreover, during high-temperature cooling, stress relaxation arising from differences in thermal expansion coefficients can also induce local delamination and bubble formation [66].

Overall, whether based on dry or wet transfer or in situ growth, the formation of interfacial bubbles are widespread during the construction of 2D material heterostructures.

### 2.3. Morphology and Chemical Composition of Bubbles

**Figure 3:**
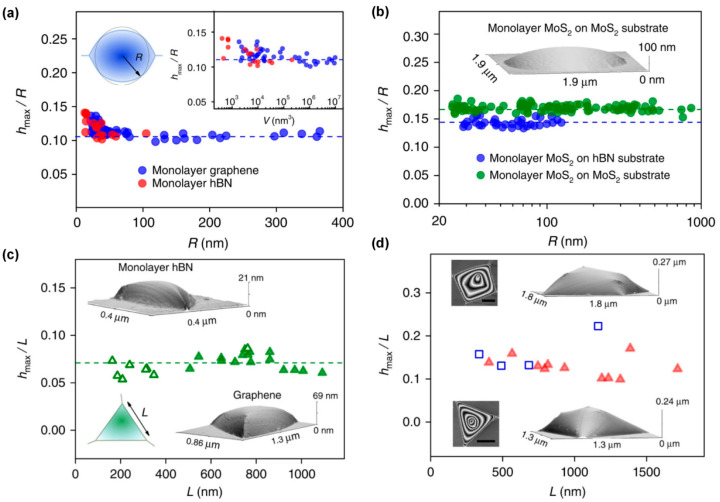
The morphology and aspect ratios of bubbles (**a**) Measured aspect ratios as a function of the base radius for graphene and monolayer hBN. Top left inset: sketch of a nearly round bubble and its effective radius R determined as *R* = $\sqrt{A/\pi }$, where *A* is the measured area of the base of the bubble. Right inset: aspect ratio of the bubbles as a function of their volume; (**b**) Aspect ratio of MoS_2_ bubbles on hBN and MoS_2_ substrates. The logarithmic scale is used to accommodate the large range of R. Inset: AFM image of a typical MoS_2_ bubble; (**c**) Symbols show the measured aspect ratios of graphene and hBN bubbles (closed and open symbols, respectively), both on hBN substrates, as a function of *L*. Bottom left inset: sketch of a triangular bubble. Its side length *L* was experimentally determined as $L=\sqrt{4A/\sqrt{3}}$. The other two insets show typical AFM images of smoothly deformed triangular bubbles; (**d**) Symbols show the measured aspect ratios of triangular (red-closed symbols) and trapezoidal (blue-open) graphene bubbles on hBN substartes, as a function of their side length, *L*, determined as $L=\sqrt{4A/\sqrt{3}}$ for triangular bubbles and as $L=\sqrt{A}$ for trapezoidal ones. Insets show typical AFM images of such bubbles: left and right are top and 3D views, respectively. Scale bars, 500 nm (Reproduced with permission from [17]).

The classical thin-film model proposed by Bunch et al. and subsequent studies by Khestanova et al. indicate that the bubble aspect ratio (h/R) remains approximately constant across a wide range of dimensions, where h and R represent the bubble height and radius, respectively [17]. The morphology and aspect ratios of the bubbles are shown in Figure 3. This size-independent aspect ratio reflects a universal mechanical behavior observed across graphene, hBN, MoS_2_, and other 2D materials, and arises from the balance between elastic stretching and vdW adhesion at the interface [4,17,67,68,69]. Bubble shape is further modulated by membrane thickness, adhesion energy, and substrate roughness; small bubbles typically approach hemispherical profiles, whereas larger bubbles become increasingly flattened due to stretching-dominated deformation [17,70]. Recent studies also demonstrate that bubble dimensions can be reversibly adjusted through external stimuli—including electric fields, mechanical strain, or ion intercalation—providing potential routes for active bubble manipulation [71].

Chemically, bubbles typically contain solvent residues, adsorbed gas molecules, transfer media, or reaction byproducts. Time-of-flight secondary ion mass spectrometry (ToF-SIMS) and XPS analyses reveal signatures of C, O, and H, indicating trapped organic moieties or oxidized contaminants [72,73]. Different fabrication routes lead to distinct internal compositions, reflecting their process-specific contamination sources.

## 3. Bubble Visualization Techniques

To understand the formation mechanism, interfacial structure, and removal effect of bubbles in 2D materials, systematic characterization methods are essential. At present, research on interfacial bubbles forms a multi-scale characterization system, ranging from macroscopic morphology observation to atomic-scale interface analysis. The main techniques include optical microscopy (OM), AFM, transmission electron microscopy (TEM), and spectroscopic analysis. This section summarizes the principles, applications, and advantages and limitations of these methods.

### 3.1. OM Method

**Figure 4:**
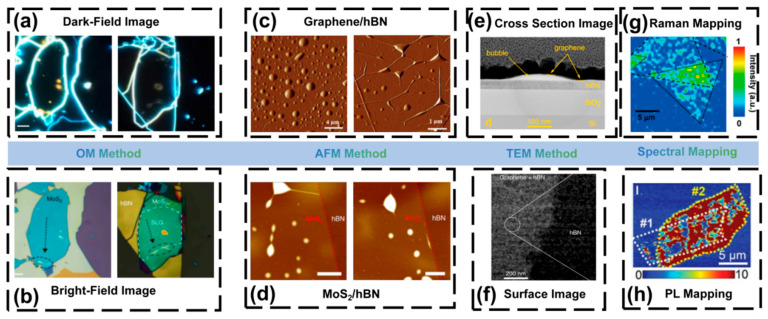
Characterization techniques of bubbles (**a**) OM characterization of bubbles in Dark-Field; (**b**) OM characterization of bubbles under Bright-Field (Reproduced with permission from [23]); (**c**) Bubbles in graphene/hBN heterostructures under AFM characterization; (**d**) Bubbles in MoS_2_/hBN heterostructures under AFM characterization (Reproduced with permission from [79,80]); (**e**) TEM characterization image of bubble cross-section; (**f**) TEM characterization image of bubble surface (Reproduced with permission from [69,74]); (**g**) Bubbles in Raman characterization; (**h**) Bubbles in PL characterization (Reproduced with permission from [81,82]).

OM is the most direct and widely used method to identify bubbles in 2D materials. Due to changes in layer thickness and optical interference, bubbles usually appear as ring-shaped contrast in optical images, allowing intuitive observation of their presence and distribution. This method enables rapid statistical analysis of bubble density, coverage fraction, and morphology on millimeter-scale areas, and is commonly used for preliminary evaluation of samples [74]. Figure 4a and Figure 4b show OM dark-field and bright-field images of bubbles, respectively.

However, the spatial resolution of OM is limited by optical diffraction, making it difficult to resolve sub-micron bubbles or small wrinkles. Moreover, bubbles and surface contamination may exhibit similar contrast; thus additional characterization methods are required for verification. Recently, image-processing approaches have been used to automatically extract bubble density and area, allowing statistical comparison across different fabrication conditions [17].

### 3.2. AFM Method

AFM is a core technique for studying interfacial bubbles in 2D materials. AFM can accurately measure the height, radius, and curvature of bubbles, and in combination with elastic membrane theory, extract interfacial adhesion energy and internal pressure [75]. Figure 4c,d show AFM images of bubbles in different 2D heterostructures. Khestanova et al. systematically studied bubbles in graphene, MoS_2_, and h-BN, and confirmed that their shapes follow general thin-film elasticity scaling laws, enabling quantitative fitting of pressure and adhesion from bubble height-radius relations [17].

In addition, PeakForce mode can obtain adhesion force and modulus distribution at bubble edges, allowing identification of interfacial residues and mechanical stability. AFM can quantitatively evaluate bubble density and size before and after treatment to determine removal efficiency [76]. Limitations include slow scan speed, limited area, and risk of puncturing bubbles if probe force is too high. Using spherical AFM tips can reduce probe-induced stress and improve measurement stability [21].

### 3.3. TEM Method

TEM can directly reveal internal composition, interface morphology, and structural features of bubbles. Haigh et al. used focused ion beam (FIB) cross-sectioning and observed bubbles in graphene/h-BN heterostructures, finding residual hydrocarbons inside bubbles, while the clean regions showed an interlayer spacing of ~0.33 nm [18]. Figure 4e,f show cross-sectional and surface TEM images. Wang et al. demonstrated that using a SiNx-supported clean assembly method can significantly reduce organic residues and achieve atomically clean interfaces based on scanning transmission electron microscopy (STEM) observations [77].

However, TEM sample preparation is complex and may introduce ion-beam damage, and the observed region is limited, which reduces statistical representativeness. Therefore, TEM is mainly used for mechanism verification and theoretical model calibration rather than large-area characterization.

### 3.4. Spectroscopic Method

Raman and photoluminescence (PL) mapping can provide strain and electronic information without physical contact. The curvature and strain caused by bubbles lead to peak shifts, linewidth broadening, and spectral intensity changes. For graphene, G and 2D peaks redshift; for TMDs (such as MoS_2_ and WSe_2_), PL peak energy decreases and linewidth broadens [19]. Raman mapping can reveal strain concentration near bubble edges. Banszerus et al. performed PL mapping on WSe_2_/h-BN heterostructures before and after annealing and observed enhanced PL intensity and reduced linewidth, indicating improved interface cleanliness after bubble removal [78]. Figure 4g,h show Raman and PL images of bubbles. Spectroscopy methods are fast and non-destructive, but the spatial resolution is diffraction-limited, which restricts precise characterization.

In practical research, individual techniques cannot fully capture bubble characteristics. High-quality studies typically employ a multi-scale combined characterization strategy: OM for detection → AFM for quantitative analysis → TEM for atomic-scale validation → Raman/PL for strain and electronic effects. Meanwhile, AI-assisted imaging analysis is increasingly adopted to enhance detection accuracy, accelerate data processing, and improve correlation across different characterization modalities [26,83,84].

## 4. Effects of Bubbles

The previous chapter systematically introduced characterization methods of interfacial bubbles. These approaches not only provide direct evidence for understanding bubble formation and evolution but also lay the foundation for evaluating their influence on the physical properties and device performance of 2D materials. After clarifying structural features and interfacial behavior, this chapter discusses the effects of bubbles on mechanical, electrical, thermal, and optical properties of 2D materials.

### 4.1. Mechanical Reliability

**Figure 5:**
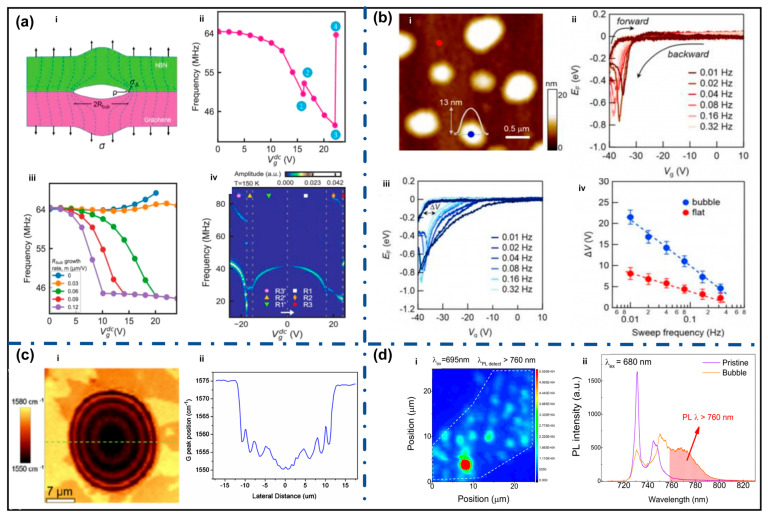
Effects of bubbles (**a**) The Effect of bubbles on the mechanical reliability of materials, i. Stress concen tration at the bubble edge, where the interfacial bubble acts as an elliptical crack producing amplified local stress, ii. Gate-induced bubble expansion causing nonlinear tension modulation and anomalous frequency dispersion, iii. Bubble-driven interlayer stick-slip events resulting in abrupt resonant frequency jumps, iv. Complete bubble-induced delamination evidenced by the emergence of new high-frequency vibration modes (Reproduced with permission from [87]); (**b**) The effect of bubbles on the electrical transport properties of materials, i. AFM topography of MoS_2_ surface showing nanoscale bubbles, ii. Frequency-dependent Fermilevel hysteresis curves measured on flat MoS_2_ region, iii. Enhanced frequency-dependent EF-hysteresis in bubble region, iv. Comparison of ΔV as a function of frequency for bubble and flat regions (Reproduced with permission from [89]); (**c**) The effect of bubbles on the thermal transport of materials, i. Raman map of the G band position of a bilayer graphene bubble, ii. G band line profile across the graphene bubble with an oscillation of the G band position (Reproduced with permission from [93]); (**d**) The effect of bubbles on the optical properties, i. Selective photoluminescence mapping of localized exciton (LX) emission in WSe_2_ using a detection window above 760 nm, ii. Photoluminescence spectra of a bubble site and a pristine region under 680 nm excitation, highlighting enhanced LX emission at λ > 760 nm (Reproduced with permission from [94]).

The mechanical reliability of 2D materials and their vdW heterostructures is significantly affected by interfacial bubbles formed during fabrication or post-processing [85,86]. Sangani et al. investigated bubble dynamics in graphene/h-BN membranes and found that when the bubble size exceeds a critical crack length, electrostatic actuation can induce irreversible interlayer delamination [87]. As shown in Figure 5a, the elliptical bubble boundary causes stress concentration similar to a crack tip, greatly amplifying local stress relative to external loading, thus weakening interfacial stability.

With continuous mechanical loading, bubble expansion leads to nonlinear modulation of membrane tension, causing anomalous frequency dispersion and mechanical response mismatch. When interfacial shear stress exceeds a critical threshold, discrete stick–slip sliding occurs, accompanied by abrupt jumps in resonant frequency, indicating fracture-like dynamic instability near bubble edges. Ultimately, delamination propagates and results in complete separation of layers, confirming bubble-induced failure. During repeated mechanical cycling or device operation, bubble edges often act as initiation sites for delamination and crack propagation. Studies show that small bubbles can grow and merge, and once energy release exceeds interfacial fracture toughness, catastrophic failure may occur suddenly [19,88]. Therefore, even sparsely distributed bubbles can significantly shorten device lifetime.

### 4.2. Electrical Transport Properties

Experimental studies show that interfacial bubbles cause local potential fluctuations and carrier scattering, reducing carrier mobility and device stability. Lee et al. demonstrated that bubbles introduce additional charge traps, altering electrical transport characteristics, as shown in Figure 5b. Compared with flat regions, bubble regions exhibit larger hysteresis under Fermi level modulation and the hysteresis width increases significantly at lower sweep frequencies, indicating slow trap dynamics [89].

Tyagi et al. reported that cleaned graphene on SiO_2_/Si exhibited hole mobility of ~9000 cm^2^/V·s and electron mobility of ~8000 cm^2^/V·s, nearly twice that of samples processed via standard acetone cleaning [90]. In recent improved wet-transfer methods, removing interfacial contamination before final transfer reduced sheet resistance and electron–hole asymmetry, implying suppressed scattering and potential fluctuations in cleaned samples [91]. Goossens et al. reported that removing surface residues decreases Dirac point shifts and background doping while significantly increasing field-effect mobility [61]. These results consistently indicate that bubbles and trapped residues form charge traps or scattering centers, reducing transport performance.

### 4.3. Thermal Transport

Bubbles alter thermal transport pathways in 2D materials by creating suspended regions that weaken interfacial heat coupling. Bacsa et al. used Raman-based thermometry to study large graphene bubbles and observed extremely high local temperatures, indicating that heat preferentially dissipates through gas pathways within bubbles rather than conducting into the substrate, leading to higher interfacial thermal resistance [92].

Huang et al. showed that during laser scanning across bubbles, Raman peak positions exhibited oscillatory changes due to local temperature gradients and abnormal heat diffusion (Figure 5c), demonstrating bubble-induced thermal pathway distortion [93]. Thus, bubbles significantly increase interfacial thermal resistance and degrade heat dissipation efficiency.

### 4.4. Optical Properties

Bubbles introduce local strain fields and interface-induced optical interference, which disrupt exciton dynamics and radiation pathways, leading to strong spatial nonuniformity in optical responses. Lee et al. observed that bubble regions in hBN-encapsulated monolayer WSe_2_ showed enhanced PL intensity and spectral shifts (Figure 5d) [94]. Similar behavior was observed in multilayer MoS_2_, where strain induced by bubbles triggered multiple direct and indirect transitions, causing strong spatial variation in linewidth and PL intensity [95]. Studies on ReS_2_/graphene heterostructures show that strain and interference jointly modulate local electric field distribution, resulting in changes in PL energy and efficiency [96].

These observations are consistent with theoretical work indicating that localized strain can drive an “exciton funneling” effect, concentrating excitons in highly strained regions and producing optical hotspots [97]. Research on pseudo-magnetic fields in graphene bubbles further supports that strong deformation reshapes band structure and density of states, indirectly affecting absorption and emission [98]. Overall, bubbles cause strain, refractive-index changes, and spatially uneven electromagnetic fields, leading to optical inhomogeneity and reduced device uniformity.

## 5. Bubble Removal and Interface Cleaning Strategies

The previous chapter outlined the adverse effects of interfacial bubbles on the mechanical, electrical, thermal, and optical properties of 2D materials, clarifying the multiple mechanisms through which they degrade performance. Given that these effects depend strongly on bubble morphology, composition, and mechanical state—and are further influenced by fabrication and post-processing conditions—the next chapter will compare key bubble removal and interface-cleaning strategies, including thermal annealing, chemical treatment, AFM cleaning, electric field-driven methods, clean assembly techniques and AI-assisted methods. We briefly discuss their applicability, working mechanisms, and practical considerations. Table 1 summarizes the principles, advantages, and limitations of these methods. AI-assisted methods are primarily used to enhance and optimize existing bubble removal techniques rather than to introduce independent physical mechanisms for bubble removal; therefore, they are not listed as a separate category in the table.

**Table 1:** Summary of bubble removal Methods.

Method	Principles	Advantages	Limitations	References
Thermal annealing	Heating drives interfacial bubbles to migrate and coalesce	Simple process; scalable; effective for most material systems	High temperature may damage materials or substrates; dependent on thermal stability	[99,100,101,102,103,104]
Chemical-assisted	Solvent wetting, gas exchange, or chemical decomposition of interfacial residues	Mild conditions; compatible with flexible substrates and sensitive devices	Possible solvent residues; limited effect on large sealed bubbles	[19,23,77,105,106,107,108,109,110,111]
AFM	AFM tip applies localized force to move and remove bubbles	High spatial precision; suitable for device-level post-processing; allows selective bubble removal in designated areas	Low efficiency; may introduce strain or mechanical damage	[22,103,112,113]
Electric field-driven	Applied voltage induces bubble motion or reduces interfacial adhesion, enabling bubble removal	No need for high temperature or solvents; can be controlled in situ	Relies on interfacial water or ions; potential electrochemical side reactions; poor uniformity; still under early development	[114,115,116]
Clean Assembly	Suppresses bubble nucleation during assembly	Enables large-area clean interfaces; prevents bubble formation at the source	High process requirements; strong dependence on specialized equipment	[43,74,117,118,119]

### 5.1. Thermal Annealing

Thermal annealing is currently one of the most commonly used and technologically mature methods for removing interfacial bubbles in 2D materials [99,100,101]. The basic principle is to heat samples in an inert atmosphere, allowing adsorbed water, solvent residues, organic contaminants, and trapped gases at the interface to gradually escape under elevated temperature, while reducing energy mismatch and adhesion barriers between the film and substrate, thereby promoting bubble migration, coalescence, and release [102,103]. After annealing, 2D materials typically exhibit higher interfacial flatness, reduced charge impurity scattering, and more stable electronic performance [4,120,121].

Early TEM studies directly observed a significant reduction in bubble volume in graphene/h-BN heterostructures after annealing, accompanied by contaminants being expelled toward the edges [18]. Subsequently, Kretinin et al. systematically proposed the concept of a “self-cleaning effect,” indicating that under thermal driving forces, contaminants spontaneously aggregate into isolated bubble regions, enabling atomically clean contact over large areas [69]. Wang et al. further confirmed that annealed interfaces can significantly enhance carrier mobility, laying the foundation for subsequent high-quality device fabrication [122].

**Figure 6:**
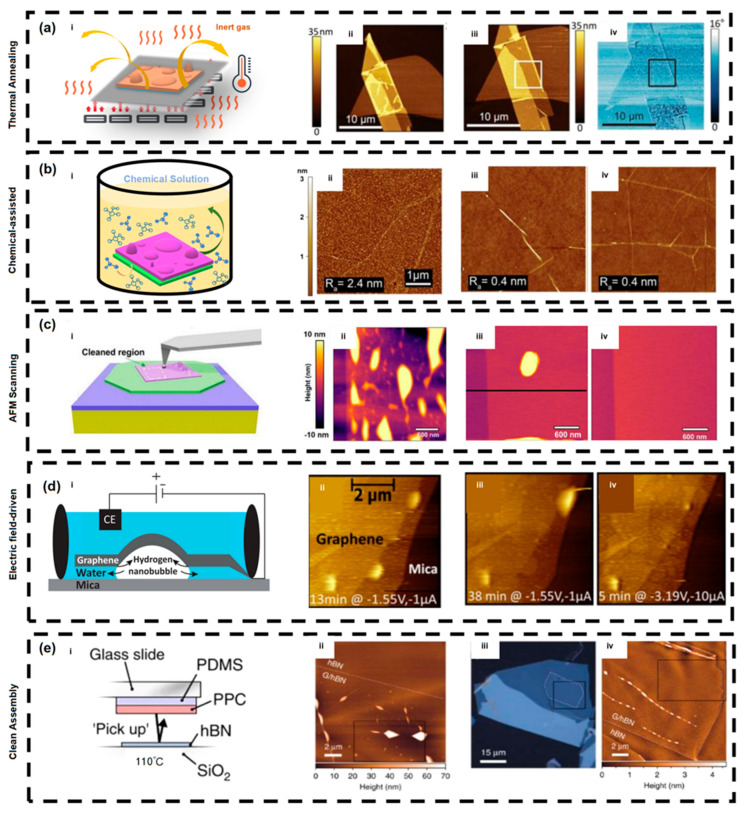
Bubble Removal Strategies (**a**) Thermal annealing method, i. Schematics of the bubble removal mechanism via thermal annealing, ii. AFM image before thermal annealing, iii–iv. AFM image after thermal annealing (Reproduced with permission from [81]); (**b**) Chemical-Assisted Method, i. Schematics of the chemical-assisted bubble removal method, ii. AFM image of the material before solution treatment, iii. AFM image of the material after HCl solution treatment, iv. AFM image of the material after NaCl solution treatment (Reproduced with permission from [145]); (**c**) AFM Method, i. Schematics of the AFM bubble removal mechanism, ii. AFM image before treatment, iii–iv. AFM image after treatment (Reproduced with permission from [21]); (**d**) Electric Field-Driven Method, i. Schematics of the electric field-driven method, ii–iv. AFM images after treatments with different electric field strengths and durations (Reproduced with permission from [114]); (**e**) Clean Assembly and Pre-treatment Methods, i. Schematics of the hot pick-up method, ii. AFM image of the sample prepared without a clean-transfer process, iii–iv. Image of the sample prepared using a clean-transfer process (Reproduced with permission from [74]).

With regard to specific process parameters, required annealing temperature and duration depend on the material system and contaminant type. For graphene/hBN heterostructures, the most commonly used conditions are 300–350 °C for 2–3 h under an Ar/H_2_ (9:1) atmosphere, which can reduce bubble area by more than 80% [18]. For TMDs such as MoS_2_ and WS_2_, due to lower thermal stability, medium-to-low temperature annealing (200–250 °C) is typically used. Lee et al. reported that annealing at 200 °C enables effective bubble removal and stabilization in hBN-encapsulated MoS_2_ devices [123]. Figure 6a shows the schematic diagram of thermal-annealing-based bubble removal and AFM images of an hBN/hBN stack before and after annealing, where bubbles are clearly reduced.

It is worth noting that despite its generality and high efficiency, thermal annealing still has limitations: high temperature may cause deformation and degradation of flexible substrates or polymer support layers [104]; bubbles that lack migration pathways may not fully escape [76]; and annealing is less effective for high-boiling-point contaminants or inert impurities such as metallic particulates [124]. To address these issues, researchers have proposed improved techniques such as multi-step thermal treatment and localized laser annealing [20,23,125,126]. The former releases interfacial adsorbates in stages and can modulate optoelectronic responses [23,125], while the latter uses localized heating-induced temperature and pressure gradients to precisely remove bubbles, demonstrated in WS_2_/hBN and graphene/SiO_2_ interfaces [127,128]. Overall, due to its simplicity, high repeatability, and broad compatibility, thermal annealing remains one of the fundamental methods for bubble removal and interface cleaning in 2D materials.

### 5.2. Chemical-Assisted Method

Chemical-assisted bubble removal modifies interfacial wettability and local chemical environments under mild conditions, thereby promoting the release of trapped gas, organic residues, or moisture from the interface between 2D materials and the substrate. Such methods are more compatible with heat-sensitive materials and flexible substrates, and can be easily integrated into existing transfer processes. Therefore, they have received broad attention in practical fabrication and device repair applications [19,105].

Common approaches for chemical-assisted bubble removal include:

1. Solvent infiltration: exposing samples to polar or semi-polar solvents or their vapor, allowing solvents to permeate along interlayer gaps and wet bubble boundaries, reducing interfacial tension and promoting bubble collapse and re-adhesion [23,77,105,106,107];
2. Gas-phase replacement: using inert or reducing gas mixtures to replace or chemically convert interfacial residues, making them easier to volatilize or disperse [108,109,110,111];
3. Chemical degradation: decomposing polymer residues under controlled atmospheres or catalytic conditions, followed by solvent removal of degradation products [129,130].

These mechanisms often occur in coupled forms: for example, after solvent infiltration reduces interfacial energy, gas release becomes easier under atmospheric replacement or mild heating. Schwartz et al. revealed the chemical composition of nanoscale contaminants in heterostructures using photo-thermal resonant spectroscopy and pointed out that solvent pretreatment and atmosphere control can significantly reduce the interfacial aggregation of such contaminants, thereby indirectly decreasing bubble formation [105]. Figure 6b presents the mechanism and effect of chemical-assisted bubble removal; after immersing graphene/SiO_2_ samples in HCl and NaCl solutions for two days, AFM scans show a large reduction in bubble density.

For bubbles of micron scale or smaller, solvent vapor treatment or gas-phase replacement can reduce residual bubble density by several tens to nearly one hundred percent; for large-scale or fully sealed blisters (>1 μm), chemical approaches often require combination with local mechanical intervention to achieve complete removal [105,131]. In addition, improper solvent control may leave trace residues or modify interfacial doping, while high-temperature gas treatment may introduce thermal stress or degradation in certain materials. Therefore, process parameters must be carefully optimized according to material systems and verified through characterization [105].

### 5.3. AFM-Based Method

Among numerous post-transfer cleaning techniques, AFM-based mechanical cleaning has become one of the most effective methods for achieving atomically clean interfaces due to its high controllability, non-destructive nature, and multifunctionality. AFM not only enables topographical imaging, but also allows precise regulation of local forces at the nanoscale, thereby enabling manipulation and removal of interfacial bubbles formed during transfer or stacking processes [22,103].

The AFM method refers to scanning the upper 2D layer with an AFM probe under controlled loading force. Through mechanical pushing or “sweeping,” contaminants at the interface are driven toward the edges, where they escape or accumulate, thus no longer affecting interface quality. The key advantages of this method lie in its non-destructive and localized operation, enabling precise cleaning without damaging the lattice. Rosenberger et al. first demonstrated the method in graphene–hBN heterostructures, resulting in samples whose optical and electronic properties approached those of ideal exfoliated layers [22]. The AFM probe acts like a nanoscale “Squeegee” redistributing and ultimately expelling interfacial bubbles. This laid the foundation for using nanomechanical control to tune vdW interfaces.

Subsequent studies further optimized probe geometry and operating parameters. Chen et al. proposed an AFM-based technique for cleaning and planarization, finding that a normal force below 100 nN effectively flattens graphene/hBN interfaces and restores high carrier mobility. Their study also reported that cleaned graphene/hBN devices exhibited room-temperature mobilities exceeding 80,000 cm^2^·V^−1^·s^−1^, approaching theoretical limits [76]. Hu’s group introduced the concept of nano spherical AFM probes and used them to sweep bubbles [113]. Ding and Qiao observed that after cleaning by nano spherical AFM probes, PL intensity increased by approximately twofold and linewidths significantly narrowed. In addition to that, the probe enabled non-destructive cleaning of soft materials such as MoS_2_ and WS_2_, successfully removing bubbles with heights below 50 nm while preserving surface integrity [112]. Figure 6c illustrates the basic principle of AFM-based bubble removal and AFM height maps before and after scanning; after scanning, the surface becomes smoother as bubbles are cleared.

From a materials engineering perspective, AFM-based bubble removal not only improves interface cleanliness, but also enhances device reliability and uniformity. Hou and Schweizer reported that removal of nanoscale bubbles reduces interfacial delamination and hotspot formation during operation [25]. In optoelectronic devices, reduced interfacial scattering directly translates to enhanced carrier diffusion and improved quantum efficiency [132]. Moreover, the method is compatible with encapsulation and flexible devices, enabling clean integration of 2D materials on polymer or dielectric substrates [77].

Recent reviews regard AFM cleaning as an important branch of broader mechanical and nanoscale cleaning techniques. In these systems, AFM serves not only as a topographical characterization tool, but also as an active interface manipulation instrument. Compared with other methods, AFM cleaning offers unique advantages: while thermal annealing or polymer-free transfer can reduce contamination, they lack local controllability. AFM cleaning enables selective post-process repair of specific device regions, making it particularly suitable for localized reprocessing of large-area devices. Researchers have recently developed arrayed AFM systems for parallel imaging; extending such approaches to interface cleaning could further enhance efficiency [133,134,135].

Despite these advancements, AFM cleaning still faces challenges. Its main limitations lie in low efficiency and long scan times for large areas. Meanwhile, a deeper understanding of force–chemical interactions between probe and material is crucial to avoid unintended strain or defect generation. Wu and Ding emphasized the importance of developing real-time feedback mechanisms to adaptively adjust loading force based on local morphology [80]. In the future, optimized probe designs, automated control, and hybrid thermal-mechanical cleaning approaches are expected to further improve the precision and speed of cleaning.

### 5.4. Electric Field-Driven Method

Electric field-driven regulation of interfacial bubbles has attracted increasing attention in recent years in the context of cleaning and interface engineering of 2D materials. Its core mechanism lies in the combined modulation of interfacial adhesion energy, electrochemical reaction kinetics, and local pressure gradients by an applied electric field. When a 2D material covers a substrate containing trace interfacial water or residual precursor molecules, applying an external voltage can induce electrochemical decomposition reactions at the interface, thereby generating nanoscale gas bubbles and altering local interlayer spacing. Dollekamp et al. directly demonstrated this in graphene–mica systems, showing that voltage application can generate reversible hydrogen nanobubbles at the interface and significantly modify local membrane adhesion states [114]. The formation and disappearance of such electrochemical bubbles are constrained by interfacial ion migration and reaction rates; therefore, bubble motion and removal can be controlled by adjusting voltage amplitude, polarity, and duration. The principle of the electric field-driven method is illustrated in Figure 6d, where bubbles can be observed to be removed by controlling electric field strength and duration.

In addition, electric fields can modulate the adsorption energy between a 2D membrane and its substrate by altering interfacial electrostatic potential differences, thereby achieving bubble removal-like effects. Related studies show that potential differences can induce local film detachment or blister formation, manifested as expansion, migration, or collapse of interfacial bubbles. For example, Macha et al. observed that pressure redistribution driven by potential differences in microcavity systems can lead to local delamination, revealing the possibility of electric field-controlled stability at 2D material–substrate interfaces [115]. This mechanism is particularly relevant for heterostructures with residual bubbles, as electric fields can reduce effective interfacial adhesion energy, allowing bubbles to migrate along paths of minimal energy.

At the nanoscale, electric fields can also manipulate bubbles via probe-induced dielectric forces, localized Joule heating, and flexoelectric responses. Scanning probe studies have shown that high electric fields can induce local blistering, interfacial detachment, and movement of bubble-like structures beneath graphene and other 2D materials, thereby providing a precise means of regulating interfacial mechanics [116]. Although these studies primarily focus on bubble formation and movement rather than direct bubble removal, the underlying physics of electric field-regulated adhesion and interfacial deformation provides theoretical foundations for designing electric field-assisted bubble elimination strategies.

Overall, electric field-driven approaches do not rely on high temperature or chemical solvents, making them suitable for in situ regulation in device-sensitive environments. Their potential limitations include the necessity of interfacial water or ions, possible adverse effects of electrochemical side reactions on materials, and challenges in uniformly applying electric fields over large areas. Current research remains concentrated on the physical processes of “electric field-induced bubble response,” and large-scale demonstrations of direct bubble removal in 2D interfaces using electric fields remain relatively scarce.

### 5.5. Clean Assembly Methods

In recent years, researchers have increasingly recognized that optimizing assembly processes to achieve clean fabrication can suppress the trapping of bubbles and contaminants at the source, significantly improving interface flatness and electrical uniformity [74,117]. Among these strategies, the hot-pickup method is one of the most representative clean assembly approaches. Pizzocchero et al. first proposed this method, in which the polymer transfer film is heated to approximately 90–110 °C during stacking. The temperature-induced interfacial flow during lamination forces bubbles and residual polymers to be expelled toward the edges, resulting in large-area bubble-free hBN/graphene/hBN heterostructures, as shown in Figure 6e [74]. This method not only enables device-level clean interfaces but also preserves interlayer coupling in subsequent fabrication, and has been widely used in preparing samples for quantum Hall and tunneling experiments. Later, Martanov et al. further standardized this technique through controlled peeling rates and alignment optimization, enabling high repeatability and laying the groundwork for scalable fabrication [118].

On this basis, Iwasaki et al. proposed an innovative “all-dry transfer” technique. By introducing hemispherical protrusions on the PDMS/PPC transfer stamp, the method achieves controlled contact with a defined contact angle, thereby suppressing bubble formation from a dynamic perspective [136]. The core of this approach lies in controlling the contact angle and speed, allowing lamination to proceed uniformly from the center outward, providing continuous escape pathways for gas and contaminants. With slow contact and moderate heating (40–55 °C), the hBN layer spreads over graphene without trapping air. AFM characterization shows that the resulting hBN/graphene/hBN samples contain no residual bubbles, with interfacial roughness below 0.2 nm. Device measurements reveal electron mobility up to 50 m^2^·V^−1^·s^−1^ and Dirac point voltages near 0 V, indicating minimal interfacial doping and scattering. Since this method does not rely on solvents or annealing, it is considered a “structure-design-based clean assembly strategy” that prevents bubble formation at the source and holds promise for clean encapsulation and large-scale manufacturing.

Another category of strategies focuses on polymer-free transfer techniques. Wang et al. used a silicon nitride (Si_3_N_4_) membrane as a temporary support, enabling direct pickup and release without organic media, thereby eliminating organic residues and bubble nucleation at the root [77]. They reported that the “ultra-clean assembly” process produced hBN/graphene interfaces with nearly no carbon residues or voids under STEM, along with more than 50% improvement in mobility. The same group later summarized the principles of polymer-free assembly, stating that by controlling surface energy matching and mechanical compliance, adsorption of water vapor and volatile residues between layers can be avoided [117].

Assembly in inert atmospheres has also proven effective. Early work by Zomer et al. showed that transferring graphene in an argon glove box significantly reduces adsorption of water and oxygen molecules from air, thereby reducing bubble nucleation density [102]. More recently, Kim et al. employed argon-shielded transfer and systematically compared interfacial morphology under air vs. inert conditions, finding that bubble density decreased by approximately 70% in the latter case, demonstrating the importance of environmental control [137].

Mechanistically, clean assembly methods prevent bubble formation or facilitate their migration by regulating interfacial energy and fluid dynamics. Temperature gradients and mechanical loading alter local interfacial stress distributions, facilitating the diffusion and removal of trapped gas or contaminants; inert environments and polymer-free systems suppress the formation of adsorbed moisture and organic residues, reducing nucleation sources. Collectively, these methods realize interfaces characterized by “low contamination, low curvature, and high adhesion energy,” forming the basis for high-performance 2D devices.

Despite significant improvements in interface quality, clean assembly techniques still face challenges. For instance, hot-pickup and pressure-controlled lamination require strict equipment and operational conditions, making large-scale integration difficult; polymer-free transfer is challenging for flexible or curved substrates; and the high cost and complexity of inert-environment operation limit broad adoption [43,119]. Future directions may include developing automated platforms with programmable pressure and temperature control, as well as surface-energy-engineered materials that enable self-adaptive bubble release, ultimately achieving industrial-scale clean assembly.

### 5.6. AI-Assisted Methods

AI has recently emerged as a valuable complementary tool for understanding and removing interfacial bubbles in vdW heterostructures. Although AI does not introduce new physical bubble removal mechanisms, it can indirectly yet effectively enhance existing strategies by enabling automated defect identification, high-throughput data analysis, fabrication parameter optimization, and closed-loop control in transfer and assembly processes. These capabilities are particularly important for improving reproducibility and accelerating the transition from device-scale demonstrations to wafer-scale integration.

#### 5.6.1. AI for Bubble Identification

As mentioned in Chapter 3, microscopy and spectroscopy are essential for bubble characterization and assessing interfacial cleanliness after transfer. Traditional manual inspection, however, is time-consuming and prone to operator bias. ML -assisted image analysis has demonstrated excellent performance in automatically detecting 2D flakes, defects, and structural features [26,138,139]. ML-based spectroscopy feature extraction further enables recognition of strain variations, contamination levels, or nanoscale heterogeneities associated with trapped bubbles [26,83,140].

Beyond microscopy and spectroscopy-based detection, neural networks have also been extended to analyze electronic transport data for bubble identification. Song et al. first demonstrated that a 2D convolutional neural network (CNN) can recognize single graphene nanobubbles by analyzing density of states (DOS) spectra, accurately predicting their geometric parameters [141]. Building upon this foundation, Kim et al. extended the approach to detect multiple nanobubbles simultaneously using a 1D CNN architecture with a unified labeling scheme, achieving 90.6% classification accuracy for up to three bubbles [142]. This approach enables rapid, purely electrical characterization, offering a complementary pathway for high-throughput bubble detection in device fabrication workflows.

Accurate and high-throughput bubble identification enabled by these ML approaches provides timely and reliable feedback on interfacial defect states, thereby greatly improving the efficiency and precision of bubble removal strategies during device fabrication.

#### 5.6.2. AI-Assisted Optimization of Preparation

As reported by Ge et al. [27], deep-learning-based optical image recognition is now used to screen exfoliated flakes by automatically identifying thickness variations, cracks, and particulate contamination, ensuring that only high-quality flakes enter the transfer workflow. During the pickup and release process, force-sensor signal analysis combined with real-time optical feedback enables the system to detect non-uniform contact—such as local lifting, excessive adhesion, or contaminated regions—and adjust temperature, pressure, or peeling speed accordingly to achieve more conformal adhesion. Moreover, process-data–driven optimization models trained on historical transfer records can recommend suitable transfer temperatures, pressures, and peeling trajectories that consistently yield cleaner interfaces. Together, these data-driven approaches enhance transfer stability and interface uniformity, thereby reducing the likelihood that residues or trapped gas pockets evolve into interfacial bubbles.

Building on recent advances in autonomous flake-searching and stacking systems [143] as well as wafer-scale robotic transfer technologies [144], AI-driven automated assembly is markedly improving interfacial quality and enabling more stable, contamination-free contact at the wafer scale, thereby effectively suppressing bubble formation.

## 6. Challenges and Outlook

### 6.1. Challenges

Although understanding of interfacial bubbles in 2D materials has deepened in recent years, current research still faces several fundamental challenges. First, how factors such as internal bubble composition, pressure state, interfacial chemistry, and stress distribution jointly influence device performance still lacks a quantitative theoretical framework, leading to an incomplete understanding of bubble-related mechanisms. Second, although various bubble removal techniques such as thermal treatment, solvent-assisted methods, AFM cleaning, and electric field-driven approaches have been proposed, the removal mechanisms of these methods themselves have not been systematically clarified. In addition, no existing approach has yet demonstrated reliable, precise, and fully effective bubble elimination at the wafer scale. Current methods remain limited by non-uniform interfacial contact, residual contamination, and constrained bubble-migration dynamics, which collectively hinder their scalability from device-sized samples to large-area integration. Achieving truly bubble-free interfaces across full wafers therefore remains a major challenge for the practical deployment of wafer-scale vdW heterostructures. Meanwhile, bubbles may undergo reconstruction, regeneration, or even chemical evolution during device operation, and their dynamic behavior and impact on long-term reliability have not yet been fully revealed. More importantly, current research remains concentrated on a limited number of typical 2D material systems, while bubble behavior in different material families and complex interfacial architectures has rarely been systematically reported.

### 6.2. Outlook

Future breakthroughs in bubble research will rely on the coordinated development of mechanistic understanding, in situ observation, and process engineering. It is necessary to establish multi-scale theoretical models that simultaneously describe adhesion, diffusion, interfacial reactions, and strain coupling, thereby revealing the full dynamic pathway of bubble formation and removal, and providing predictive guidance for material selection, process design, and interface engineering.

AI is expected to play an increasingly central role in advancing bubble removal strategies for 2D materials. With the rapid development of deep learning, reinforcement learning, and multimodal imaging analysis, AI can enable precise identification, tracking, and prediction of bubble evolution, while optimizing key process parameters—including transfer temperature, polymer stamp viscoelasticity, loading/unloading speeds, annealing windows, pressure profiles, ambient conditions, and electrostatic or mechanical post-treatments. By transforming empirical adjustments into data-driven and adaptive optimization, AI-enabled process control is poised to significantly enhance the efficiency, reproducibility, and cross-material applicability of existing bubble removal methods.

In progressing toward wafer-scale integration, the key challenge will shift from improving individual bubble removal techniques to embedding these methods into a fully automated, continuous, and large-area manufacturing framework. By integrating robotic manipulation platforms, high-uniformity lamination systems, and AI-guided process regulation, localized bubble removal approaches may evolve into regionally controlled and globally consistent wafer-scale strategies. Incorporating real-time interfacial monitoring, spatially resolved stress management, intelligent annealing planning, and dynamic correction mechanisms can further ensure uniform bubble removal across large areas. As AI models continue to mature under complex, multi-parameter operating conditions, a next-generation wafer-scale assembly paradigm appears increasingly within reach.

Furthermore, although bubbles have traditionally been regarded as undesired defects in 2D materials and most efforts have focused on their elimination, they are not universally detrimental. A growing body of work has shown that, under specific conditions, bubbles can be deliberately created and engineered as functional structures for probing and tuning the properties of 2D materials. For example, He et al. demonstrated the formation of controllable hydrogen-filled bubbles in hBN via plasma treatment [146], and Tedeschi et al. employed low-energy proton irradiation to induce monolayer-thick nanobubbles on the surface of bulk transition metal dichalcogenides without introducing structural defects [147]. These intentionally engineered bubbles enable tailored local strain fields and the modulation of electronic structure, optical response, and chemical reactivity [19,85,148,149,150]. The existence of such engineered bubble systems provides a more balanced perspective on the multifaceted roles of bubbles in vdW heterostructures, highlighting that bubbles may act not only as imperfections to be removed but also as functional elements under appropriate conditions.

## Data Availability

No new data were created or analyzed in this study. Data sharing is not applicable to this article.
